# Immunotherapy combined with definitive chemoradiotherapy for locally advanced unresectable esophageal squamous cell carcinoma

**DOI:** 10.3389/fimmu.2025.1646568

**Published:** 2025-09-30

**Authors:** Yidan Hong, Qishu Tan, Yixin Li, Bo Yang, Yinan Wu, Lijun Zhao, Yang Zhao, Yu Chen, Zihao Zhu, Xiangzhi Zhu, Lingling Gu, Ning Jiang

**Affiliations:** ^1^ Department of Radiation Oncology, The Affiliated Cancer Hospital of Nanjing Medical University, Nanjing, China; ^2^ Department of Medical Imaging Center, The Affiliated Cancer Hospital of Nanjing Medical University, Nanjing, China; ^3^ Department of Pathology, The Affiliated Cancer Hospital of Nanjing Medical University, Nanjing, China; ^4^ Department of Information, The Affiliated Cancer Hospital of Nanjing Medical University, Nanjing, China

**Keywords:** esophageal squamous cell carcinoma, immunotherapy, chemoradiotherapy (CRT), survival analysis, toxicity

## Abstract

**Background:**

The clinical value of immune checkpoint inhibitors (ICIs) in the treatment of unresectable locally advanced esophageal squamous cell carcinoma (LA-ESCC) remains under investigation in large-scale randomized clinical trials. In this study, we aimed to assess the efficacy and safety of concurrent ICIs in combination with definitive chemoradiotherapy (dCRT) in a relatively large cohort of patients with unresectable LA-ESCC.

**Methods:**

Between January 2019 and December 2023, this retrospective study included patients with LA-ESCC who received ICIs concurrently with dCRT at Jiangsu Cancer Hospital. The primary endpoints were overall survival (OS) and progression-free survival (PFS), while secondary endpoints included clinical response and safety.

**Results:**

A total of 165 patients with LA-ESCC were included in this study, with a median age of 66 years (IQR 60–70 years), and 163 (98.8%) had stage III or IVA disease. After a median follow-up of 24 months (IQR 16–33 months), the 2-year OS was 64.3% (95%CI 57.2%-72.3%), and the 2-year PFS was 50.2% (95%CI 42.9%-58.8%). It is noteworthy that induction therapy before dCRT did not improve OS (HR = 1.09, 95% CI: 0.61–1.93, *P* = 0.770) or PFS (HR = 1.00, 95% CI: 0.59–1.72, *P* = 1.000). The objective response rate (ORR) was 70.9% and disease control rate (DCR) was 86.7%. The most common adverse event was grade 3 or worse lymphopenia, observed in 60.0% of the patients (90/165).

**Conclusion:**

ICIs combined with concurrent dCRT demonstrated promising efficacy and manageable safety in patients with unresectable LA-ESCC.

## Introduction

Esophageal cancer is the sixth leading cause of cancer-related mortality globally (1). Esophageal squamous cell carcinoma (ESCC) is the predominant histological subtype in Asia, accounting for approximately 90% of all cases (2). Definitive chemoradiotherapy (dCRT) is the standard treatment for patients with unresectable locally advanced esophageal squamous cell carcinoma (LA-ESCC) (3). However, the three-year survival rate under this treatment regimen ranges between 26.9% and 55.4% (4–8), with approximately half of the patients experiencing recurrence within three years (4–6). Therefore, it is imperative to develop novel therapeutic strategies to improve patient outcomes and reduce recurrence rates.

Recently, immune checkpoint inhibitors (ICIs) have demonstrated promising applications in the treatment of esophageal cancer. In patients with advanced or metastatic ESCC, the latest findings from the Keynote 590 (9) and ESCORT-1st (10) trials have demonstrated improved clinical outcomes, establishing ICIs as part of the standard treatment regimen. Moreover, in unresectable LA-ESCC, several studies (10, 11) have also reported encouraging efficacy of ICIs when combined with dCRT concurrently, although these findings are based on relatively small patient cohorts. These studies demonstrate potential clinical benefits with improved overall survival (OS) and progression-free survival (PFS) outcomes compared to chemotherapy alone, with manageable treatment-related toxicity. Taken together, current evidence indicates that concurrent ICIs combined with dCRT may represent a promising therapeutic strategy for patients with unresectable LA-ESCC.

However, large-scale randomized phase III trials evaluating ICIs combined with dCRT concurrently for unresectable LA-ESCC remain under investigation. In this context, we conducted a retrospective analysis of LA-ESCC patients treated with this combination therapy at Jiangsu Cancer Hospital to evaluate its efficacy and safety profile.

## Methods

### Participants

This retrospective cohort study included patients aged 18–75 years diagnosed with ESCC. The inclusion criteria were as follows: (1) pathologically confirmed ESCC, (2) histological classification as II-IVA stage according to the 8^th^ TNM staging system of the American Joint Committee on Cancer (12), and (3) unsuitable for surgery due to unresectable disease, surgical contraindications, or patient refusal. The exclusion criteria were as follows: (1) presence of secondary malignancies, (2) incomplete radiotherapy, (3) prior antitumor treatment for any malignancy, and (4) patients who underwent surgery after the completion of treatment. This study was conducted according to the Declaration of Helsinki and was approved by the Ethics Committee of Jiangsu Cancer Hospital (Approval No. KY-2024-067).

### Procedures

Pretreatment evaluations included physical examinations, standard hematological and biochemical tests, pretreatment upper gastrointestinal endoscopy and biopsy, barium esophagograms, contrast-enhanced neck and upper abdominal computed tomography (CT), and high-resolution 3.0 -T magnetic resonance imaging (MRI) of the chest and brain. Ultrasonography of the neck with fine-needle aspiration and positron emission tomography/CT (PET-CT) were performed optionally for clinical tumor–lymph node–metastasis staging.

Based on primary tumor size and clinical stage, a subset of patients received induction therapy prior to the initiation of concurrent ICIs and dCRT, which was defined as completion of at least one treatment cycle of immunotherapy, chemotherapy, or their combination before radiotherapy. All patients underwent concurrent immunotherapy combined with dCRT (during and within a half-month period before and after dCRT (13, 14)). The ICIs administered were PD-1 inhibitors, and the chemotherapy regimen consisted of a taxane combined with a platinum-based agent, administered every three weeks for two cycles. For patients who were intolerant to intravenous chemotherapy, oral S-1 was used as an alternative regimen.

All patients were treated with intensity-modulated radiotherapy (IMRT) using 6-MV X-rays, receiving conventionally fractionated radiotherapy at 5 fractions per week. The gross tumor volume (GTV), which included the primary tumor (GTVp) and metastatic lymph nodes (GTVn), was determined using endoscopy, contrast-enhanced thoracic CT, and/or 18-fluorodeoxyglucose PET-CT. The primary clinical target volume (CTVp) included the GTVp with an additional radial margin of 0.5–1.0 cm and longitudinal margins of 2.5–3 cm. The clinical target volume for metastatic lymph nodes (CTVn) was delineated based on the involved field irradiation, excluding elective nodal irradiation. The planning target volume (PTV) was defined by adding a margin of 0.5–1.0 cm to the CTV. Cone-beam CT was used for daily verification during the first week of treatment, followed by weekly verification after that. The dose constraints for the organs at risk were as follows: a mean lung dose of < 17 Gy, a lung V20 (percentage of the total lung volume receiving ≥ 20 Gy) of < 30%, lung V5 (percentage of the total lung volume receiving ≥ 5 Gy) of < 65%, a mean heart dose of 26 Gy or lower, heart V30 (percentage of the total heart volume receiving ≥ 30 Gy) of < 35%, and maximum spinal cord dose of 45 Gy or lower.

### Follow-up and assessments

The primary endpoints were OS (the time from the first treatment until death from any cause or censored at the last follow-up) and PFS (the time from the first treatment until tumor progression in any aspect or death from any cause or censored at the last follow-up). The secondary evaluation endpoints were clinical response and safety.

The clinical response of the tumor was independently evaluated by two experienced oncologists within three months after the completion of radiotherapy, using the Response Evaluation Criteria in Solid Tumors (RECIST, version 1.1) (15). Treatment failures were categorized as either locoregional or distant disease, based on CT scans confirmed by pathology or identified by the investigator and senior radiologist through integration with other imaging examinations. Deaths were confirmed through official statements. Adverse events were evaluated according to the Common Terminology Criteria for Adverse Events (CTCAE, version 5.0) (16) during and up to 90 days post-completion of concurrent chemoradiotherapy. The highest score was recorded as the patient’s toxicity grade.

### Statistical methods

Continuous variables are presented as median with range or mean with standard deviations, while categorical variables are presented as frequencies with percentages. Continuous variables were compared using the *t*-test. Survival was estimated using the Kaplan–Meier method. The log-rank test was employed to evaluate the significance of survival differences between groups based on baseline characteristics, and Cox proportional hazards regression models were used to estimate the hazard ratio (HR) and 95% confidence interval (CI). To account for multiple comparisons in univariate Cox regression analysis, the Benjamini-Hochberg procedure was applied to adjust *P*-values, controlling the false discovery rate (FDR). Variables with adjust *P* < 0.2 in the univariate analysis were included in the multivariate analysis. We assessed the proportional hazards (PH) assumption for the multivariable Cox regression models of OS and PFS using Schoenfeld residuals with the cox.zph function in R. A global test *P*-value > 0.05 was considered as no evidence of PH assumption violation. Immunotherapy cycles were analyzed separately by treatment phase. Induction and maintenance phases were grouped as none, short-term, or long-term. The concurrent phase was classified according to the actual number of cycles received.

Patients were retrospectively classified according to whether they received induction therapy. To address baseline imbalances, propensity scores were estimated using logistic regression with clinically relevant covariates (age, sex, smoking, alcohol use, hypertension, diabetes mellitus, body mass index (BMI), tumor length, tumor location, and TNM stage) that may influence both treatment allocation and survival. Propensity score matching (PSM) was performed with a 1:1 nearest-neighbor algorithm (caliper = 0.1). As a sensitivity analysis, inverse probability of treatment weighting (IPTW) was also applied using the same set of covariates. Balance was evaluated using standardized mean differences (SMDs), with values <0.1 considered indicative of good balance and values between 0.1 and 0.2 regarded as acceptable. Finally, weighted Cox proportional hazards models with robust standard errors were fitted to evaluate the impact of induction therapy on OS and PFS. All statistical analyses were performed using R (version 4.4.1), with a two-sided *P* < 0.05 considered statistically significant.

## Results

### Patient characteristics

**Figure 1:**
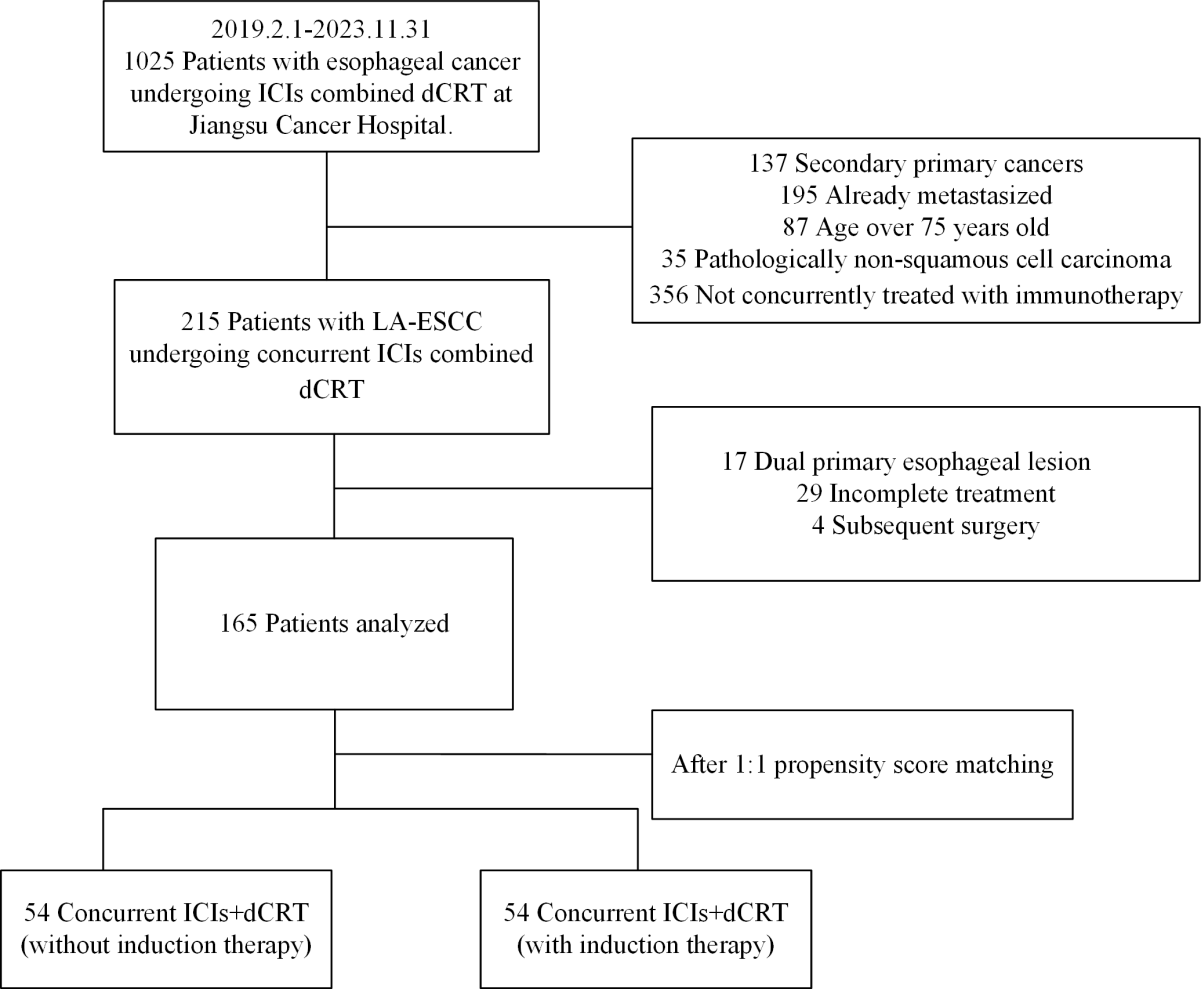
Trial profile. dCRT, definitive chemoradiotherapy.

From January 1, 2019 to December 31, 2023, 165 patients treated with dCRT and concurrent ICIs were retrospectively included in this study (**Figure 1**). Patient characteristics are described in **Table 1**. The median age was 66 years (IQR 60–70 years). Among these patients, 81 (49.1%) had stage III disease, and 82 (49.7%) had stage IVA. Clinical T4 disease was observed in 94 patients (57.0%), while 74 (44.8%) had N2 or higher nodal involvement. A total of 66 individuals (40.0%) had a tumor length of > 5 cm, with a median tumor length of 4.8 cm (IQR 3.8–6.3 cm). Tumors located in the upper esophagus were identified in 74 cases (44.8%). Based on whether received induction therapy before dCRT, patients were stratified into induction and concurrent treatment groups. Following 1:1 PSM, 54 matched pairs were obtained, and most baseline covariates achieved good balance, with SMDs <0.1. BMI showed a slightly higher SMD of 0.130, which remained within the acceptable threshold of 0.2. In the IPTW analysis, age (SMD = 0.171) and tumor location (SMD = 0.117) showed modest residual imbalance, but all covariates were within acceptable limits. Detailed covariate balance before and after adjustment is presented in **Supplementary Tables 1** and **2**.

**Table 1:** Patient characteristics.

Characteristics	Patients (n=165)
Median age (IQR), years	66 (60,70)
Sex	
Female	32 (19.4%)
Male	133 (80.6%)
BMI (kg/m2)	
<18.5	18 (10.9%)
18.5-24.9	110 (66.7%)
>24.9	37 (22.4%)
Tumor Location	
Upper	74 (44.8%)
Middle	73 (44.2%)
Distal	18 (10.9%)
Primary Tumor Length (cm)	
Median (IQR)	4.8 (3.8,6.3)
≤5	99 (60.0%)
>5	66 (40.0%)
Clinical T Stage	
T2	12 (7.3%)
T3	59 (35.8%)
T4a	42 (25.5%)
T4b	52 (31.5%)
Clinical N Stage	
N0	3 (1.8%)
N1	88 (53.3%)
N2	55 (33.3%)
N3	19 (11.5%)
Clinical TNM Stage	
II	2 (1.2%)
III	81 (49.1%)
IVA	82 (49.7%)
Smoking history	
Yes	22 (13.3%)
No	143 (86.7%)
Drinking history	
Yes	22 (13.3%)
No	143 (86.7%)

Data are n (%). All patients were Chinese. IQR, interquartile range

### Treatment

In this study, the median total number of immunotherapy cycles was 4 (range, 1–26), including a median of 0 cycles (range, 0–5) in the induction phase, 2 cycles (range, 1–3) in the concurrent phase, and 1 cycle (range, 0–25) in the maintenance phase. A total of 73 patients (44.3%) received induction ICIs prior to concurrent ICIs plus dCRT, all patients received concurrent programmed cell death protein 1 (PD-1) inhibitor therapy during dCRT, and 103 patients (62.4%) received maintenance ICIs after concurrent ICIs plus dCRT (**Table 2**). All patients received a median radiation dose of 60 Gy (IQR 50.4-60Gy) with single-fraction doses ranging from 1.8 to 2 Gy. The majority of patients (135/165, 81.8%) received taxane-platinum combination chemotherapy (**Supplementary Table 3**).

**Table 2:** Distribution of Immunotherapy Types and Treatment Cycles.

Variable	Category	N (%)	Median cycles (range)
ICIs types	Camrelizumab	64 (38.8)	–
	Sintilimab	41 (24.8)	–
	Tislelizumab	31 (18.8)	–
	Toripalimab	29 (17.6)	–
Total ICIs cycles	All patients	165 (100)	4 (1 - 26)
Induction ICIs cycles prior to concurrent ICIs + dCRT	All patients	165 (100)	0 (0 – 5)
	None	92 (55.7)	–
	1–2 cycles	48 (29.1)	–
	≥3 cycles	25 (15.2)	–
Concurrent ICIs cycles during dCRT	1 cycle	63 (38.2)	–
	2 cycles	79 (47.9)	–
	3 cycles	23 (13.9)	–
Maintenance ICIs cycles after concurrent ICIs + dCRT	All patients	165 (100)	1 (0 – 25)
	None	62 (37.6)	–
	1–4 cycles	58 (35.1)	–
	≥5 cycles	45 (27.3)	–

ICIs, immune checkpoint inhibitors; dCRT, definitive chemoradiotherapy

### Survival

**Figure 2:**
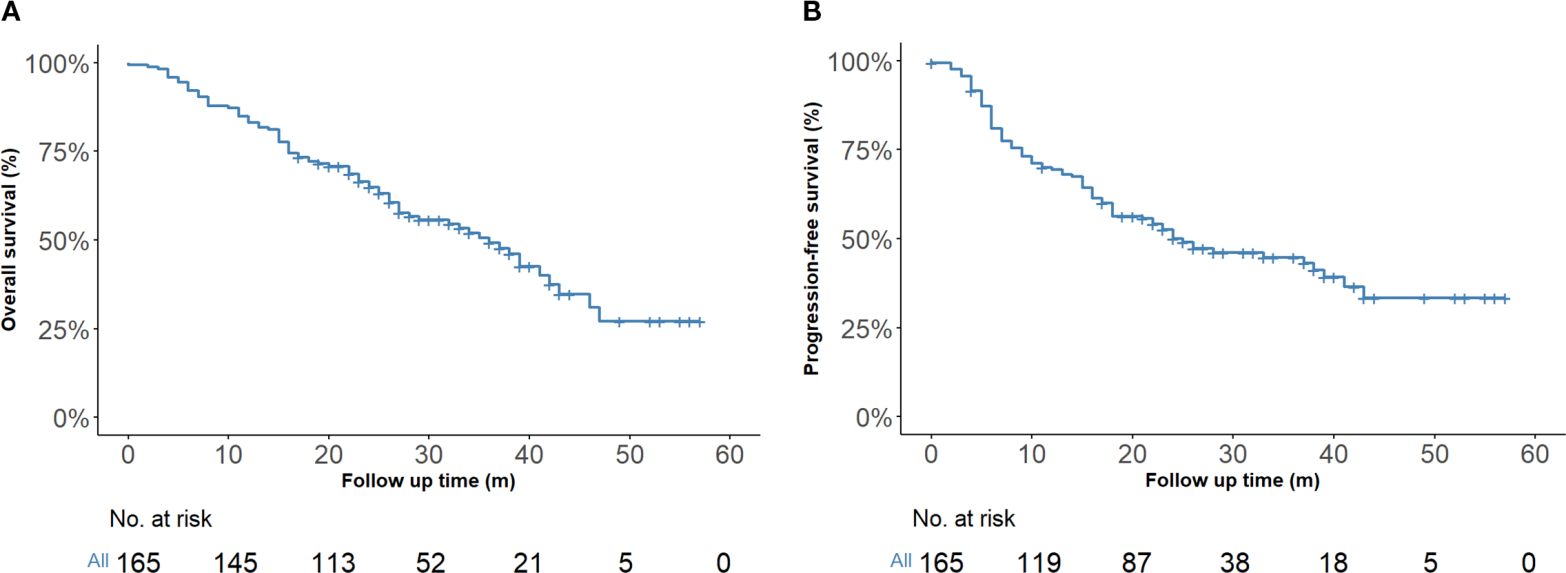
Kaplan–Meier survival estimates (n = 165). **(A)** Overall survival of the entire cohort. **(B)** Progression-free survival of the entire cohort.

By the last follow-up on March 1, 2025, the entire cohort had a median follow-up of 24 months (IQR, 16–33 months), the one-year and two-year OS rates were 83.0% (95% CI, 77.5%–89.0%) and 64.3% (95% CI, 57.2%–72.3%), respectively. The corresponding PFS rates were 69.4% (95% CI, 62.6%–76.8%) at one year and 50.2% (95% CI, 42.9%–58.8%) at two years (**Figures 2A, B**).

**Figure 3:**
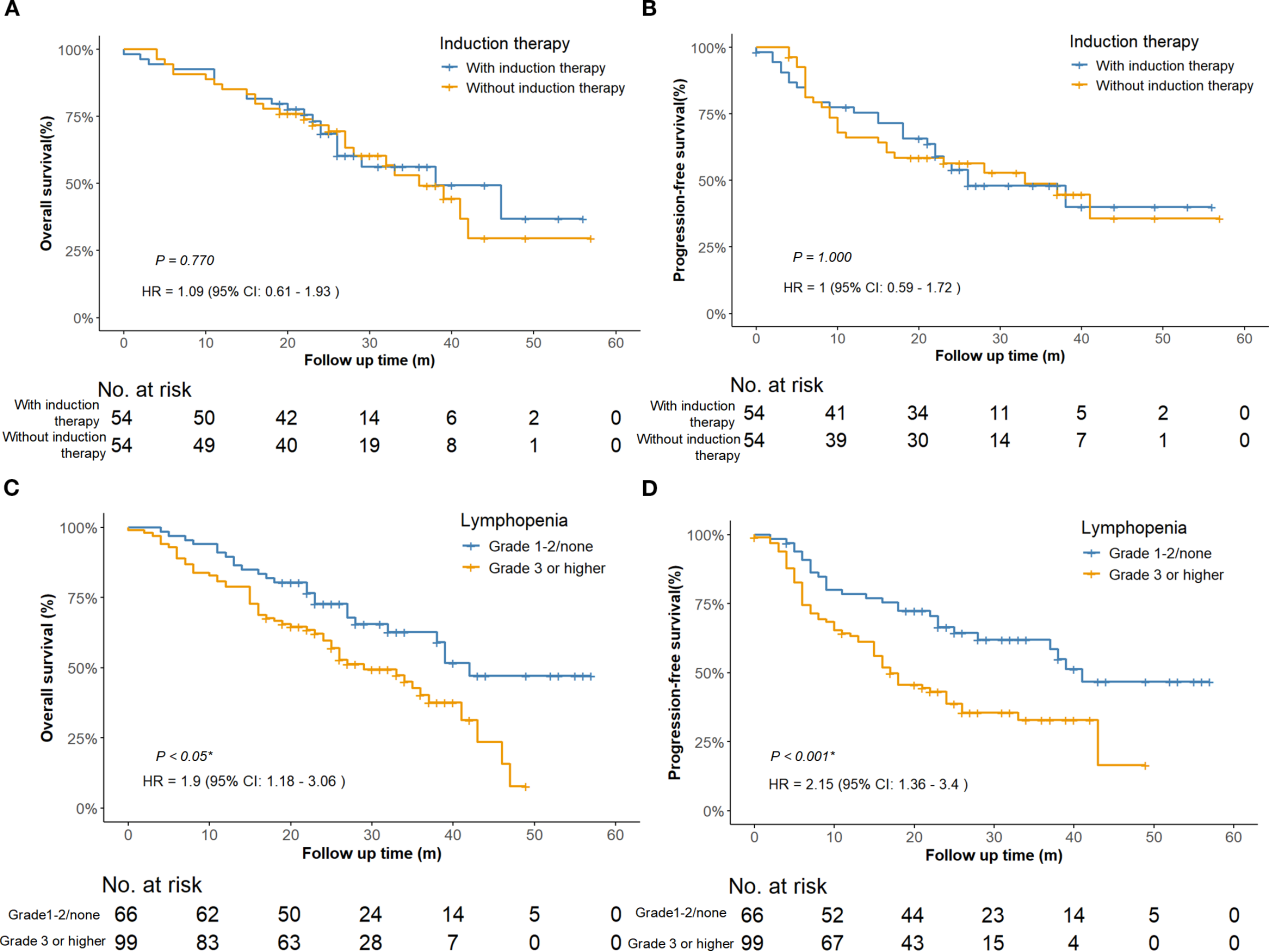
Survival curves comparing patients with or without induction therapy after PSM adjustment. (n = 108) **(A)** Overall survival. **(B)** Progression-free survival. Survival curves comparing patients with lymphopenia of Grade 1–2 or none to those with Grade 3 or higher (n = 165). **(C)** Overall survival of the entire cohort. **(D)** Progression-free survival of the entire cohort. PSM, propensity score matching; HR, hazard ratio; CI, confidence interval.

Survival analysis stratified by induction therapy status demonstrated that adding induction treatment to concurrent dCRT with ICIs had no significant impact on OS (HR = 1.09, 95% CI: 0.61–1.93, *P* = 0.770) or PFS (HR = 1.00, 95% CI: 0.59–1.72, *P* = 1.000) after PSM (**Figures 3A, B** **).** Consistently, IPTW-adjusted Cox models showed no significant survival difference between groups for either OS (HR = 1.10, 95% CI: 0.65–1.83, *P* = 0.728) or PFS (HR = 1.00, 95% CI: 0.61–1.63, *P* = 0.995), as shown in **Supplementary Figures 1A, B**. In addition, after adjustment for multiple comparisons, no significant survival differences were observed among different ICIs. Similarly, no significant associations were found between the number of immunotherapy cycles in any treatment phase and OS or PFS (**Supplementary Tables 4A**, **B** **).**

### Clinical responses

165 patients were included in the clinical response evaluation. Among the 165 patients, 54 (32.7%) achieved a complete response (CR), 63 (38.2%) achieved a partial response (PR), 26 (15.8%) maintained stable disease (SD), and 22 (13.3%) experienced progressive disease (PD). The objective response rate (ORR) was 117/165 (70.9%), and the disease control rate (DCR) was 143/165 (86.7%).

### Toxicity

All patients experienced treatment-related adverse events of any grade (**Table 3**). The most common grade 3 or higher adverse events were lymphopenia (99/165, 60.0%) and leukopenia (27/165, 16.3%). The incidence of esophagitis was 39.9%, with 2 (1.2%) cases graded as 3. Grade 1–2 pneumonitis occurred in 67 patients (40.6%), and grade 1–2 esophageal fistula in 9 (5.5%). No treatment-related deaths occurred.

**Table 3:** Treatment-related adverse events (n=165).

	Any Grade	Grade ≥ 3
Lymphopenia	152 (92.1%)	99 (60.0%)
Oesophagitis	54 (32.7%)	2 (1.2%)
Oesophageal fistula	9 (5.5%)	0
Anorexia	38 (23.0%)	0
Anaemia	75 (45.5%)	6 (3.6%)
Fatigue	2 (1.2%)	0
Cough	65 (39.4)	0
Pneumonitis	67 (40.6%)	0
Leukopenia	91 (55.1%)	27 (16.3%)
Nausea or vomiting	120 (72.7%)	0
ALT elevation	9 (5.4%)	1 (0.6%)
GGT elevation	26 (15.7%)	1 (0.6%)
ALP elevation	10 (6.1%)	1 (0.6%)
Constipation	35 (21.2%)	0
Neutropenia	44 (26.6%)	13 (7.8%)
Hypothyroidism	7 (4.2%)	0
Thrombocytopenia	80 (48.4%)	7 (4.2%)
Rash	2 (1.2%)	0
Arrhythmia	6 (3.6%)	0
Hypertriglyceridaemia	35 (21.2%)	2 (1.2%)
Fever	29 (17.6%)	0
Diarrhoea	14 (8.5%)	0
Bilirubin elevation	4 (2.4%)	1 (0.6%)
Creatinine increased	4 (2.4%)	0

Data are n (%). ALT, alanine aminotransferase; GGT, gamma-glutamyl transferase; ALP, alkaline phosphatase.

### Prognostic factors

**Figure 4:**
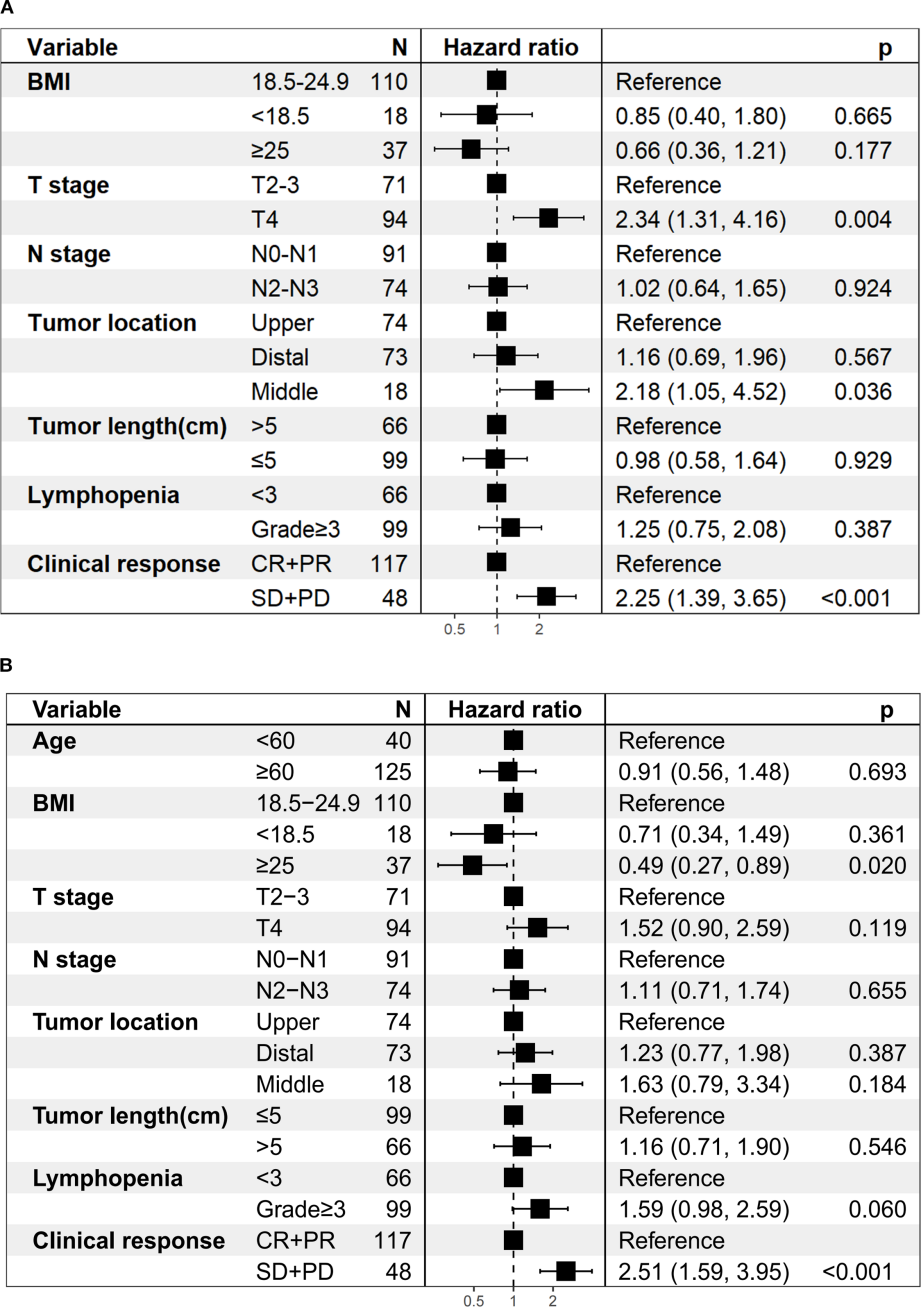
Multivariate regression analysis of prognostic factors. **(A)** Overall survival. **(B)** Progression-free survival.

Univariate analysis revealed that T stage, tumor length, lymphopenia, and clinical response were associated with both OS and PFS. In addition, tumor location was associated with OS, while BMI and N stage were related to PFS. After Benjamini–Hochberg adjustment, T stage, lymphopenia, and clinical response remained significantly associated with both OS and PFS, whereas tumor length and BMI also reached statistical significance for PFS (**Supplementary Tables 5A**, **B**). Notably, grade ≥3 lymphopenia, the most frequent toxicity observed in our study, was significantly associated with worse OS (HR = 1.90; 95% CI 1.18–3.06; *P* < 0.05; **Figure 3C**) and PFS (HR = 2.15; 95% CI 1.36–3.40; *P* < 0.001; **Figure 3D**). However, the association between lymphopenia and survival outcomes was not statistically significant in multivariate analysis. Multivariate analysis further confirmed that T4 stage (HR = 2.34, 95%CI: 1.31–4.16, *P* = 0.004), middle esophageal tumor location (HR = 2.18, 95%CI: 1.05–4.52, *P* = 0.036), and SD/PD response (HR = 2.25, 95%CI: 1.39–3.65, *P* < 0.001) were independent predictors of worse OS (**Figure 4A**). For PFS, BMI ≥25 was identified as a protective factor (HR = 0.49, 95%CI: 0.27–0.89, *P* = 0.020), whereas SD/PD response remained an independent risk factor for disease progression (HR = 2.51, 95%CI: 1.59–3.95, *P* < 0.001) (**Figure 4B**). Notably, induction therapy prior to dCRT did not confer a survival benefit in terms of PFS or OS (*P* > 0.05). The proportional hazards assumption was tested for both the OS and PFS multivariable Cox models. For the OS model, χ² = 5.412 (df = 9, *P* = 0.800), and for the PFS model, the global test yielded χ² = 13.742 (df = 10, *P* = 0.190), indicating no violation of the PH assumption. All individual covariates also showed non-significant *P*-values (> 0.05), confirming that the Cox regression models were appropriate for our data. Detailed results for individual covariates are provided in **Supplementary Table 6**.

## Discussion

This retrospective study evaluated the strategy of dCRT combined with concurrent ICIs in a relatively large cohort of patients with unresectable LA-ESCC. The one-year and two-year OS rates were 83.0% (95% CI, 77.5%–89.0%) and 64.3% (95% CI, 57.2%–72.3%), respectively. The corresponding PFS rates were 69.4% (95% CI, 62.6%–76.8%) at one year and 50.2% (95% CI, 42.9%–58.8%) at two years. This study also showed manageable treatment-related toxicity with no treatment-related deaths. Additionally, the induction therapy prior to dCRT demonstrated no survival benefit. While its application in real-world settings requires further investigation and should be guided by clinical judgment. In conclusion, the combination of dCRT with concurrent ICIs demonstrates potential as a therapeutic strategy for unresectable LA-ESCC, though its efficacy needs to be validated through large-scale randomized controlled trials.

Our findings suggest that combining dCRT with concurrent ICIs may improve survival outcomes, representing a promising therapeutic strategy for LA-ESCC patients. In our study, the addition of ICIs to concurrent dCRT resulted in a two-year OS rate of 64.3% (95% CI, 57.2%–72.3%) and a two-year PFS rate of 50.2% (95% CI, 42.9%–58.8%). Consistent with two-year OS rates ranging from 50% to 66.7% and PFS rates between 46.4% and 54.4% for standard dCRT regimens (4, 5, 17). The EC-CRT-001 phase II trial (11) evaluating concurrent ICIs with dCRT for LA-ESCC reported a promising 1-year OS rate of 78.4%, though the 1-year PFS rate (54.5%) did not exceed standard dCRT regimen. The investigators attributed this unexpected PFS outcome to their higher enrollment of stage IVA patients (38%) compared to previous studies. Notably, our cohort included a substantially greater proportion of stage IVA disease (49.7%) than reported in prior studies (8.5%-38.7%) (4, 5, 11, 17), yet still demonstrated encouraging survival outcomes, suggesting potential therapeutic benefit across advanced disease stages. Consistent with our findings, Zhang et al. (18) conducted a phase 1b study of camrelizumab combined with concurrent dCRT and reported 2-year OS and PFS rates of 69.6% and 65.0% respectively. Additional supportive evidence includes a durvalumab plus tremelimumab regimen, which achieved 2-year OS and PFS rates of 75% and 57.5% (19). A recent multicenter real-world study involving 102 ESCC patients treated with concurrent ICIs and dCRT reported 1- and 2-year OS rates of 86.7% and 66.9%, and PFS rates of 66.7% and 47.3%, respectively (20), which are generally consistent with our findings. Several ongoing phase III trials, such as KEYNOTE-975 (pembrolizumab), RATIONALE-311 (tislelizumab), KUNLUN (durvalumab), and ESCORT-CRT (camrelizumab), are expected to provide high-level evidence to further define the role of concurrent ICIs combined with dCRT in this setting. It is also important to note that the present study was conducted in a real-world clinical setting, where treatment adherence and patient management may vary from the strictly regulated conditions of prospective clinical trials. Despite these potential sources of variability, the survival outcomes observed in our study underscore the clinical value of integrating ICIs into dCRT regimens for unresectable LA-ESCC patients, even for patients with advanced-stage disease. These results provide further support for the application of immunotherapy in this population and contribute to the growing body of real-world evidence on its effectiveness.

In the era of ICIs, induction immunochemotherapy before dCRT has not been incorporated into standard guidelines for LA-ESCC, due to insufficient high-level evidence and potential toxicity concern. Large primary tumor volume has been identified as a poor prognostic factor in ESCC treated with definitive CCRT (21). Tumor volume reduction before CCRT may improve local control and survival outcomes. Phase II studies (22, 23) of this novel induction immunochemotherapy regimen have demonstrated promising improvements in local control rates and survival. These clinical benefits are biologically supported by preclinical validation of related mechanisms, including tumor vascular normalization and hypoxia alleviation (24). However, these regimens carry toxicity burdens, a high proportion of patients (80%–90%) experienced grade 3 or higher treatment-related adverse events, and cases of grade 5 pneumonitis have been reported (23). In our study, while no treatment-related deaths occurred, induction therapy combined with dCRT did not confer a survival benefit in patients with ESCC, as measured by either OS (*P* = 0.770, **Figure 3A**) or PFS (*P* = 1.000, **Figure 3B**). This difference may be attributed to the relatively small sample size in our study, as only 72 patients (43.6%) received induction immunochemotherapy, potentially limiting the statistical power to detect the therapeutic benefits of induction therapy. In conclusion, while our findings suggest induction therapy may offer limited clinical benefit in esophageal cancer management, further investigations are warranted to definitively establish its therapeutic role and fully characterize potential toxicities.

Several studies have shown that patients achieving CR after dCRT have significantly better survival than those with residual disease (7, 25). Consistent with these findings, our results demonstrated that the CR + PR group exhibited a longer OS (HR = 2.25; 95% CI 1.39–3.65; *P* < 0.001; **Figure 4A**) and PFS (HR = 2.51; 95% CI 1.59–3.95; *P* < 0.001; **Figure 4B**) compared to the SD + PD group. These findings underscore that early tumor shrinkage following dCRT combined with ICIs represents not only a marker of treatment sensitivity but also an independent prognostic indicator. Clinically, this highlights the value of early response evaluation, as patients who fail to achieve CR or PR face substantially higher risks of disease progression and mortality, and may require closer surveillance or consideration of additional systemic therapy. From a mechanistic perspective, radiotherapy can trigger the release of tumor antigens and activate antigen-presenting cells, and when combined with PD-1/PD-L1 blockade, it enhances cancer cell immunogenicity and improves long-term antitumor responses (26, 27). When considering overall response, the ORR rate in our study was 70.9%, comparable with that reported in previous studies using dCRT alone (70.9% versus 65%–74.6%) (6, 7), despite the higher disease burden in our cohort. In earlier reports, the proportion of T3–T4 patients was 72.7% (7), whereas our study included 92.7%. Similarly, stage IV patients accounted for only 10% in previous studies (6), compared with 49.7% in our cohort. To conclude, despite enrolling a high proportion of patients with advanced disease, our study achieved promising ORR and survival outcomes. This suggests that combining concurrent ICIs with dCRT not only improves tumor response but also turns these responses into real survival benefits, supporting the therapeutic value of this combined approach.

In our study, a BMI ≥25 was identified as an independent protective factor for PFS (HR = 0.49, 95% CI: 0.27–0.89, *P* = 0.020; **Figure 4B**), suggesting that patients with higher BMI may derive greater benefit from dCRT combined with immunotherapy. Although obesity is generally recognized as a risk factor for the development of several cancers (28), increasing evidence indicates that overweight (BMI 25–29) and obese (BMI ≥ 30) patients may experience improved outcomes with ICIs (29–31), a phenomenon known as the obesity paradox (32). This has been partially explained by previous preclinical studies showing that obesity-associated metabolic signalling and inflammatory cues cause tumour-associated macrophages (TAMs)to induce PD-1 expression, which then drives a TAM-specific feedback mechanism that impairs tumour immune surveillance. This may contribute to increased cancer risk yet improved response to PD-1 immunotherapy in obesity (33). Nevertheless, only 37 patients (22.4%) in our cohort had a BMI ≥25, and no cases of severe obesity (BMI >35) were included. Thus, while our findings highlight a potentially important prognostic role for BMI in this treatment setting, they should be interpreted with caution and warrant confirmation in larger prospective studies.

T4 stage was also identified as an independent adverse prognostic factor for OS (HR = 2.34, 95% CI: 1.31–4.16, *P* = 0.04; **Figure 4A**), underscoring the prognostic importance of local invasion into adjacent structures. Chen et al. found that tracheal/bronchial invasion and ulcerative tumors were independent risk factors for esophagotracheal fistula after dCRT (34). Historically, T4b disease has been associated with poor outcomes, with a 3-year OS of only 22.2% and locoregional failure in 60% of cases (35). In the immunotherapy era, the EPOC1802 trial, which included nearly 95% of patients with cT4 LA-ESCC, reported that maintenance ICIs after dCRT improved response rates and intermediate-term survival, even among patients with a high T4 tumor burden (36). Notably, in our study, despite a high proportion of T4 cases (94/165, 57%), the combination of dCRT and concurrent ICIs yielded encouraging survival outcomes without an increased incidence of post-dCRT esophagotracheal fistula, suggesting that immunotherapy integration may confer benefit with acceptable safety in this high-risk population. Prospective validation is warranted.

The prognostic impact of tumor location in the setting of dCRT remains understudied, and existing evidence is inconsistent. Gao et al (37). reported that tumor location was independently associated with OS and PFS in multivariate analysis, with upper thoracic tumors achieving the highest 5-year OS (46.0%). This suggests that middle and lower thoracic tumors may have worse outcomes, possibly due to their proximity to critical structures such as the heart, lungs, and great vessels, which can limit radiation dose delivery and increase the risk of severe cardiopulmonary complications. Other dCRT studies found no statistically significant association between location and survival but noted a trend toward poorer outcomes for upper/middle thoracic disease compared with lower thoracic disease (*P* = 0.055) (38). In our cohort, middle esophageal tumor location was independently associated with worse OS (HR = 2.18, 95% CI: 1.05–4.52, *P* = 0.036; **Figure 4A**), supporting the clinical value of incorporating tumor location into individualized target delineation and dose optimization. However, the number of patients with middle esophageal tumors was small (18/165, 10.9%), which may limit the statistical power and highlights the need for validation in larger, multicenter cohorts.

In our study, 60% of patients developed grade ≥ 3 lymphopenia, including 20 cases (12.1%) of grade 4, with no grade 5 events observed. Lymphocytes are highly radiosensitive, with significant depletion occurring even at relatively low radiation doses (39). This suggests that the observed lymphopenia is more likely attributable to radiotherapy rather than ICIs. Thoracic radiation, especially when involving large planning target volumes or vertebral bone marrow, has been shown to induce profound lymphocyte loss (40). Mechanistic studies have further demonstrated that radiation not only reduces circulating lymphocytes and suppresses lymphopoiesis, but also upregulates immune inhibitory molecules such as PD-L1 and CTLA-4, contributing to systemic immunosuppression (41, 42). Similar patterns of lymphopenia have been reported in previous studies of dCRT without the use of ICIs. Davuluri et al. observed a 27% incidence of grade 4 lymphopenia in esophageal cancer patients treated with dCRT alone (43), and Deng et al. reported grade 3–4 lymphopenia in up to 50% of patients receiving thoracic IMRT (44). These findings support the safety and feasibility of concurrently integrating ICIs into dCRT without significantly increasing severe lymphopenia. Moreover, severe lymphopenia has been associated with inferior survival outcomes across multiple studies (43–45). In our univariate analysis, patients with grade ≥3 lymphopenia had significantly shorter overall survival (HR = 1.90; 95% CI: 1.18–3.06; *P* < 0.05; **Figure 3C**) and progression-free survival (HR = 2.15; 95% CI: 1.36–3.40; *P* < 0.001; **Figure 3D**). Although these associations were not statistically significant in the multivariate analysis, the findings raise the possibility that lymphopenia may negatively impact clinical outcomes. Importantly, the combination of ICIs with dCRT did not appear to worsen treatment-related lymphopenia, further supporting its potential as a safe therapeutic strategy.

Our study has certain limitations. As this was a single-center, retrospective study, the generalizability of our findings is inevitably limited. The sample size was relatively small, and the median follow-up duration was only 24 months, which may hinder a thorough evaluation of long-term survival outcomes and delayed toxicities, including the potential long-term impact of immune-related adverse events. Future larger prospective studies with extended follow-up are needed to provide a more robust evaluation of long-term efficacy and safety. Although PSM was performed to minimize confounding, the retrospective and single-center nature of this study may still introduce selection bias. Therefore, our findings should be interpreted with caution, and validation through prospective, multi-center randomized trials will be essential to confirm the reliability and generalizability of our findings. In addition, the inability to standardize the type of PD-1 inhibitors could have introduced variability in treatment response. The assessment of clinical response was primarily based on CT imaging, which may compromise the accuracy of short-term efficacy evaluation. Further use of EUS and PET/CT in future studies could be integral in identifying regional nodes and assessing the risk of metastasis. Since this study was conducted in a single hospital within a real-world clinical setting, treatment decisions were made by clinicians based on routine practice, potentially influencing the observed outcomes and mirroring the practical complexities inherent in everyday patient management. In addition to the primary endpoints of OS and PFS, further research should include a comprehensive analysis of PD-L1 expression and the role of the tumor microenvironment in predicting treatment efficacy.

## Conclusion

Our study provides preliminary evidence that the combination of dCRT with concurrent ICIs offers promising survival benefits for patients with unresectable LA-ESCC, while maintaining a manageable safety profile. However, phase III randomized controlled trials are warranted to validate these findings and further establish the role of this treatment strategy.

## Data Availability

The data analyzed in this study is subject to the following licenses/restrictions: The raw data supporting the conclusions of this article will be made available by the authors, without undue reservation. Requests to access these datasets should be directed to Jiang Ning, njiang117@njmu.edu.cn.
