# Supplementary material for: Immunotherapy combined with definitive chemoradiotherapy for locally advanced unresectable esophageal squamous cell carcinoma
Source: Front Immunol. 2025 Sep 30;16:1646568. doi: 10.3389/fimmu.2025.1646568 (PMC12518077; doi:10.3389/fimmu.2025.1646568)
Supplement: Supplementary file 1 [file DataSheet1.docx]

**Supplementary table 1:** Patient characteristics with standardized mean differences before and after propensity score matching

| Characteristics |  | Before PSM | | | After PSM | | |
| --- | --- | --- | --- | --- | --- | --- | --- |
|  |  | Concurrent ICIs+dCRT  (without induction therapy) | Concurrent ICIs+dCRT  (with induction therapy) | SMD | Concurrent ICIs+dCRT  (without induction therapy) | Concurrent ICIs+dCRT  (with induction therapy) | SMD |
|  |  | N=62 | N=103 |  | N=54 | N=54 |  |
| Age(%) | <60years | 5 (8.1) | 35 (34.0) | 0.671 | 5 (9.3) | 5 (9.3) | <0.001 |
|  | ≥60years | 57 (91.9) | 68 (66.0) |  | 49 (90.7) | 49 (90.7) |  |
| Sex(%) | Female | 15 (24.2) | 17 (16.5) | 0.192 | 13 (24.1) | 12 (22.2) | 0.044 |
|  | Male | 47 (75.8) | 86 (83.5) |  | 41 (75.9) | 42 (77.8) |  |
| Smoking (%) | No | 55 (88.7) | 88 (85.4) | 0.098 | 49 (90.7) | 50 (92.6) | 0.067 |
|  | Yes | 7 (11.3) | 15 (14.6) |  | 5 (9.3) | 4 (7.4) |  |
| Alcohol (%) | No | 57 (91.9) | 86 (83.5) | 0.259 | 49 (90.7) | 50 (92.6) | 0.067 |
|  | Yes | 5 (8.1) | 17 (16.5) |  | 5 (9.3) | 4 (7.4) |  |
| Hypertension (%) | No | 44 (71.0) | 71 (68.9) | 0.044 | 39 (72.2) | 40 (74.1) | 0.042 |
|  | Yes | 18 (29.0) | 32 (31.1) |  | 15 (27.8) | 14 (25.9) |  |
| Diabetes mellitus (%) | No | 56 (90.3) | 95 (92.2) | 0.068 | 48 (88.9) | 48 (88.9) | <0.001 |
|  | Yes | 6 (9.7) | 8 (7.8) |  | 6 (11.1) | 6 (11.1) |  |
| BMI (%) | <18.5 | 11 (17.7) | 7 (6.8) | 0.343 | 6 (11.1) | 4 (7.4) | 0.130 |
|  | ≥25 | 12 (19.4) | 25 (24.3) |  | 12 (22.2) | 13 (24.1) |  |
|  | 18.5-24.9 | 39 (62.9) | 71 (68.9) |  | 36 (66.7) | 37 (68.5) |  |
| Tumor length (%) | >5cm | 22 (35.5) | 44 (42.7) | 0.149 | 18 (33.3) | 18 (33.3) | <0.001 |
|  | ≤5cm | 40 (64.5) | 59 (57.3) |  | 36 (66.7) | 36 (66.7) |  |
| Tumor location (%) | Distal | 28 (45.2) | 45 (43.7) | 0.443 | 25 (46.3) | 24 (44.4) | 0.038 |
|  | Middle | 2 (3.2) | 16 (15.5) |  | 2 (3.7) | 2 (3.7) |  |
|  | Upper | 32 (51.6) | 42 (40.8) |  | 27 (50.0) | 28 (51.9) |  |
| TNM (%) | II-III | 36 (58.1) | 47 (45.6) | 0.251 | 32 (59.3) | 32 (59.3) | <0.001 |
|  | VA | 26 (41.9) | 56 (54.4) |  | 22 (40.7) | 22 (40.7) |  |

PSM = propensity score matching, SMD = standardized mean differences

dCRT = definitive chemoradiotherapy, BMI = Body Mass Index

**Supplementary Tables 2:** Patient characteristics with standardized mean differences before and after IPTW analysis

|  | **Before IPTW** | | | **After IPTW** | | |
| --- | --- | --- | --- | --- | --- | --- |
| Variable | (n=62 / 103) | *P*-value | SMD | (n=154.2 / 164.8) | *P*-value | SMD |
| **Age** |  |  |  |  |  |  |
| ≥60, n (%) | 57 (91.9) / 68 (66.0) | <0.001 | 0.671 | 127.7 (82.8) / 125.1 (76.0) | 0.434 | 0.171 |
| **Sex** |  |  |  |  |  |  |
| Male, n (%) | 47 (75.8) / 86 (83.5) | 0.314 | 0.192 | 124.1 (80.4) / 132.6 (80.5) | 0.992 | 0.002 |
| **Smoking** |  |  |  |  |  |  |
| Yes, n (%) | 7 (11.3) / 15 (14.6) | 0.717 | 0.098 | 19.8 (12.9) / 22.6 (13.7) | 0.888 | 0.026 |
| **Alcohol** |  |  |  |  |  |  |
| Yes, n (%) | 5 (8.1) / 17 (16.5) | 0.191 | 0.259 | 17.0 (11.0) / 22.6 (13.7) | 0.662 | 0.082 |
| **Hypertension** |  |  |  |  |  |  |
| Yes, n (%) | 18 (29.0) / 32 (31.1) | 0.920 | 0.044 | 46.0 (29.8) / 51.0 (31.0) | 0.89 | 0.025 |
| **Diabetes mellitus** |  |  |  |  |  |  |
| Yes, n (%) | 6 (9.7) / 8 (7.8) | 0.890 | 0.068 | 16.9 (10.9) / 16.3 (9.9) | 0.86 | 0.035 |
| **BMI category** |  | 0.087 | 0.343 |  | 0.929 | 0.064 |
| <18.5 | 11 (17.7) / 7 (6.8) |  |  | 16.4 (10.6) / 14.9 (9.0) |  |  |
| 18.5–24.9 | 39 (62.9) / 71 (68.9) |  |  | 100.3 (65.0) / 111.5 (67.7) |  |  |
| ≥25 | 12 (19.4) / 25 (24.3) |  |  | 37.5 (24.3) / 38.3 (23.3) |  |  |
| **Tumor length** |  |  |  |  |  |  |
| ≤5 cm, n (%) | 40 (64.5) / 59 (57.3) | 0.450 | 0.149 | 94.3 (61.1) / 99.4 (60.3) | 0.927 | 0.017 |
| **Tumor location** |  | 0.041 | 0.443 |  | 0.831 | 0.117 |
| Upper | 32 (51.6) / 42 (40.8) |  |  | 65.8 (42.7) / 71.7 (43.5) |  |  |
| Middle | 2 (3.2) / 16 (15.5) |  |  | 12.1 (7.8) / 18.0 (10.9) |  |  |
| Distal | 28 (45.2) / 45 (43.7) |  |  | 76.3 (49.5) / 75.1 (45.6) |  |  |
| **TNM stage VA, n (%)** | 26 (41.9) / 56 (54.4) | 0.166 | 0.251 | 68.9 (44.7) / 79.8 (48.4) | 0.682 | 0.074 |

IPTW = inverse probability of treatment weighting; SMD = standardized mean differences; dCRT = definitive chemoradiotherapy; BMI = Body Mass Index

Supplementary table 3: Chemotherapy regimens

| Regimens | Patients(n=165) |
| --- | --- |
| Taxane +platinum | 135(81.8%) |
| Taxane | 11(6.7%) |
| S-1 | 19(11.5%) |

**Supplementary Table 4A.** Overall Survival According to Immunotherapy Type and Treatment Cycle Number

| **Characteristic** | **N** | **HR** | **95% CI** | ***P*-value** | **Adj.**  ***P*-value** |
| --- | --- | --- | --- | --- | --- |
| **Immunotherapy** |  |  |  |  |  |
| Camrelizumab | 64 | — | — |  |  |
| Sintilimab | 41 | 0.80 | 0.43, 1.49 | 0.485 | 0.939 |
| Tislelizumab | 31 | 1.20 | 0.66, 2.21 | 0.548 | 0.939 |
| Toripalimab | 29 | 1.19 | 0.67, 2.14 | 0.553 | 0.939 |
| **Induction Immunotherapy** |  |  |  |  |  |
| None | 92 | — | — |  |  |
| 1-2 cycles | 48 | 0.98 | 0.58, 1.65 | 0.939 | 0.939 |
| >2 cycles | 25 | 1.52 | 0.85, 2.69 | 0.158 | 0.939 |
| **Concurrent Immunotherapy** |  |  |  |  |  |
| 1 cycle | 63 | — | — |  |  |
| 2 cycles | 79 | 1.06 | 0.66, 1.72 | 0.811 | 0.939 |
| 3 cycles | 23 | 0.85 | 0.43, 1.69 | 0.649 | 0.939 |
| **Maintenance Immunotherapy** |  |  |  |  |  |
| None | 62 | — | — |  |  |
| 1-4 cycles | 58 | 1.05 | 0.62, 1.77 | 0.860 | 0.939 |
| >4 cycles | 45 | 0.83 | 0.48, 1.44 | 0.502 | 0.939 |

HR = hazard ratio, CI = confidence interval

Adj. *P*-value = *P*-values adjusted using the Benjamini–Hochberg procedure

**Supplementary Table 4B.** Progression-Free Survival According to Immunotherapy Type and Treatment Cycle Number

| **Characteristic** | | **N** | **HR** | **95% CI** | ***P*-value** | **Adj.**  ***P*-value** |
| --- | --- | --- | --- | --- | --- | --- |
| **Immunotherapy** | |  |  |  |  |  |
| Camrelizumab | | 64 | — | — |  |  |
| Sintilimab | | 41 | 0.73 | 0.42, 1.28 | 0.274 | 0.913 |
| Tislelizumab | | 31 | 0.79 | 0.44, 1.45 | 0.452 | 0.913 |
| Toripalimab | | 29 | 1.05 | 0.60, 1.85 | 0.853 | 0.913 |
| **Induction Immunotherapy** | |  |  |  |  |  |
| None | | 92 | — | — |  |  |
| 1-2 cycles | | 48 | 0.95 | 0.58, 1.55 | 0.835 | 0.913 |
| >2 cycles | | 25 | 1.49 | 0.86, 2.60 | 0.159 | 0.913 |
| **Concurrent Immunotherapy** | |  |  |  |  |  |
| 1 cycle | | 63 | — | — |  |  |
| 2 cycles | | 79 | 1.17 | 0.74, 1.85 | 0.495 | 0.913 |
| 3 cycles | | 23 | 1.16 | 0.61, 2.18 | 0.651 | 0.913 |
| **Maintenance Immunotherapy** | |  |  |  |  |  |
| None | | 62 | — | — |  |  |
| 1-4 cycles | | 58 | 1.03 | 0.62, 1.69 | 0.913 | 0.913 |
| >4 cycles | | 45 | 1.18 | 0.70, 1.97 | 0.541 | 0.913 |
|  |  |  |  |  |  |  |

HR = hazard ratio, CI = confidence interval

Adj. *P*-value = *P*-values adjusted using the Benjamini–Hochberg procedure

**Supplementary table 5A:** Univariate and Multivariate Cox Regression Analysis of Factors Associated with Overall Survival (Raw and Adjusted *P*-values)

| **Characteristic** | **Univariable** | | | |  | **Multivariable** | | |
| --- | --- | --- | --- | --- | --- | --- | --- | --- |
|  | **HR^1^** | **95% CI^1^** | | ***P*-value^2^** | **Adj. *P*-value^2^** | **HR^1^** | **95% CI^1^** | ***P* -value^2^** |
| **Age** |  |  | |  |  |  |  |  |
| ˂60 | — | — | |  |  |  |  |  |
| ≥60 | 0.83 | 0.51, 1.35 | | 0.444 | 0.622 |  |  |  |
| **Sex** |  |  | |  |  |  |  |  |
| Female | — | — | |  |  |  |  |  |
| Male | 0.82 | 0.48, 1.40 | | 0.465 | 0.622 |  |  |  |
| **Smoking** |  |  | |  |  |  |  |  |
| No | — | — | |  |  |  |  |  |
| Yes | 0.96 | 0.51, 1.83 | | 0.911 | 0.951 |  |  |  |
| **Alcohol** |  |  | |  |  |  |  |  |
| No | — | — | |  |  |  |  |  |
| Yes | 0.80 | 0.40, 1.60 | | 0.532 | 0.655 |  |  |  |
| **Hypertension** |  |  | |  |  |  |  |  |
| No | — | — | |  |  |  |  |  |
| Yes | 1.25 | 0.78, 2.00 | | 0.358 | 0.622 |  |  |  |
| **Diabetes mellitus** |  |  | |  |  |  |  |  |
| No | — | — | |  |  |  |  |  |
| Yes | 0.92 | 0.42, 2.01 | | 0.837 | 0.951 |  |  |  |
| **BMI** |  |  | |  |  |  |  |  |
| 18.5-24.9 | — | — | |  |  | — | — |  |
| ˂18.5 | 0.98 | 0.48, 1.98 | | 0.952 | 0.951 | 0.85 | 0.40, 1.80 | 0.665 |
| ≥25 | 0.58 | 0.32, 1.05 | | 0.072 | 0.157 | 0.66 | 0.36, 1.21 | 0.177 |
| **T stage** |  |  | |  |  |  |  |  |
| T2-3 | — | — | |  |  | — | — |  |
| T4 | 2.32 | 1.43, 3.77 | | <0.001*** | 0.005** | 2.34 | 1.31, 4.16 | 0.004** |
| **N stage** |  |  | |  |  |  |  |  |
| N0-N1 | — | — | |  |  | — | — |  |
| N2-N3 | 1.48 | 0.96, 2.30 | | 0.079 | 0.157 | 1.02 | 0.64, 1.65 | 0.924 |
| **Tumor length(cm)** |  |  | |  |  |  |  |  |
| ≤5 | — | — | |  |  | — | — |  |
| ˃5 | 1.73 | 1.11, 2.69 | | 0.015* | 0.060 | 1.02 | 0.61, 1.72 | 0.929 |
| **Tumor location** |  |  | |  |  |  |  |  |
| Upper | — | — | |  |  | — | — |  |
| Distal | 1.55 | 0.95, 2.51 | | 0.077 | 0.157 | 1.16 | 0.69, 1.96 | 0.567 |
| Middle | 2.06 | 1.05, 4.05 | | 0.037* | 0.117 | 2.18 | 1.05, 4.52 | 0.036* |
| **Induction therapy** |  |  | |  |  |  |  |  |
| No | — | — | |  |  |  |  |  |
| Yes | 1.19 | 0.75, 1.89 | | 0.466 | 0.622 |  |  |  |
| **Lymphopenia** |  |  | |  |  |  |  |  |
| ˂3 | — | — | |  |  | — | — |  |
| Grade≥3 | 1.90 | 1.18, 3.06 | | 0.009** | 0.045* | 1.25 | 0.75, 2.08 | 0.387 |
| **Clinical response** |  |  | |  |  |  |  |  |
| CR+PR | — | — | |  |  | — | — |  |
| SD+PD | 2.40 | 1.53, 3.79 | | <0.001*** | 0.003** | 2.25 | 1.39, 3.65 | <0.001*** |
|  | | | ^1^HR = Hazard Ratio, CI = Confidence Interval | | | | | |
|  | | | ^2^*p<0.05; **p<0.01; ***p<0.001 | | | | | |

Adj. *P*-value = *P*-values adjusted using the Benjamini–Hochberg procedure

CR = complete response, PR = partial response, SD = stable disease, PD = progressive disease

**Supplementary table 5B:** Univariate and Multivariate Cox Regression Analysis of Factors Associated with Progression-Free Survival

(Raw and Adjusted *P*-values)

| **Characteristic** | **Univariable** | | | |  | **Multivariable** | | |
| --- | --- | --- | --- | --- | --- | --- | --- | --- |
|  | **HR^1^** | **95% CI^1^** | | ***P*-value^2^** | **Adj. *P*-value^2^** | **HR^1^** | **95% CI^1^** | ***P* -value^2^** |
| **Age** |  |  | |  |  |  |  |  |
| ˂60 | — | — | |  |  | — | — |  |
| ≥60 | 0.64 | 0.40, 1.00 | | 0.052 | 0.118 | 0.91 | 0.56, 1.48 | 0.693 |
| **Sex** |  |  | |  |  |  |  |  |
| Female | — | — | |  |  |  |  |  |
| Male | 1.03 | 0.60, 1.74 | | 0.924 | 0.924 |  |  |  |
| **Smoking** |  |  | |  |  |  |  |  |
| No | — | — | |  |  |  |  |  |
| Yes | 1.06 | 0.58, 1.95 | | 0.853 | 0.910 |  |  |  |
| **Alcohol** |  |  | |  |  |  |  |  |
| No | — | — | |  |  |  |  |  |
| Yes | 0.82 | 0.43, 1.59 | | 0.558 | 0.646 |  |  |  |
| **Hypertension** |  |  | |  |  |  |  |  |
| No | — | — | |  |  |  |  |  |
| Yes | 1.22 | 0.78, 1.90 | | 0.383 | 0.613 |  |  |  |
| **Diabetes mellitus** |  |  | |  |  |  |  |  |
| No | — | — | |  |  |  |  |  |
| Yes | 0.80 | 0.37, 1.73 | | 0.566 | 0.646 |  |  |  |
| **BMI** |  |  | |  |  |  |  |  |
| 18.5-24.9 | — | — | |  |  | — | — |  |
| ˂18.5 | 0.80 | 0.40, 1.60 | | 0.522 | 0.646 | 0.71 | 0.34, 1.49 | 0.361 |
| ≥25 | 0.47 | 0.26, 0.86 | | 0.015* | 0.047* | 0.49 | 0.27, 0.89 | 0.020* |
| **T stage** |  |  | |  |  |  |  |  |
| T2-3 | — | — | |  |  | — | — |  |
| T4 | 1.87 | 1.20, 2.91 | | 0.006** | 0.031* | 1.52 | 0.90, 2.59 | 0.119 |
| **N stage** |  |  | |  |  |  |  |  |
| N0-N1 | — | — | |  |  | — | — |  |
| N2-N3 | 1.57 | 1.03, 2.37 | | 0.035* | 0.093 | 1.11 | 0.71, 1.74 | 0.655 |
| **Tumor length(cm)** |  |  | |  |  |  |  |  |
| ≤5 | — | — | |  |  | — | — |  |
| ˃5 | 1.73 | 1.14, 2.62 | | 0.010** | 0.040* | 1.16 | 0.71, 1.90 | 0.546 |
| **Tumor location** |  |  | |  |  |  |  |  |
| Upper | — | — | |  |  | — | — |  |
| Distal | 1.36 | 0.87, 2.13 | | 0.182 | 0.323 | 1.23 | 0.77, 1.98 | 0.387 |
| Middle | 1.77 | 0.92, 3.41 | | 0.090 | 0.179 | 1.63 | 0.79, 3.34 | 0.184 |
| **Induction therapy** |  |  | |  |  |  |  |  |
| No | — | — | |  |  |  |  |  |
| Yes | 1.18 | 0.76, 1.83 | | 0.459 | 0.646 |  |  |  |
| **Lymphopenia** |  |  | |  |  |  |  |  |
| ˂3 | — | — | |  |  | — | — |  |
| Grade≥3 | 2.15 | 1.36, 3.40 | | 0.001** | 0.008** | 1.59 | 0.98, 2.59 | 0.060 |
| **Clinical response** |  |  | |  |  |  |  |  |
| CR+PR | — | — | |  |  | — | — |  |
| SD+PD | 2.72 | 1.78, 4.17 | | <0.001*** | <0.001*** | 2.51 | 1.59, 3.95 | <0.001*** |
|  | | | ^1^HR = Hazard Ratio, CI = Confidence Interval, | | | | | |
|  | | | ^2^*p<0.05; **p<0.01; ***p<0.001 | | | | | |

Adj. *P*-value = *P*-values adjusted using the Benjamini–Hochberg procedure

CR = complete response, PR = partial response, SD = stable disease, PD = progressive disease

**Supplementary Table 6.** Schoenfeld residuals test for proportional hazards assumption in the Cox models for OS and PFS

|  |  | OS |  |  | PFS |  |
| --- | --- | --- | --- | --- | --- | --- |
| **Covariate** | **χ²** | **df** | ***P*-value** | **χ²** | **df** | ***P*-value** |
| Age | — | — | — | 0.669 | 1 | 0.410 |
| BMI | 4.596 | 2 | 0.100 | 3.088 | 2 | 0.210 |
| T stage | 0.228 | 1 | 0.630 | 0.538 | 1 | 0.460 |
| N stage | 0.316 | 1 | 0.570 | 2.309 | 1 | 0.130 |
| Tumor location | 0.129 | 2 | 0.940 | 1.38 | 2 | 0.500 |
| Tumor length | 0.455 | 1 | 0.500 | 1.422 | 1 | 0.230 |
| Lymphopenia | 0.058 | 1 | 0.810 | 0.128 | 1 | 0.720 |
| Clinical response | 0.036 | 1 | 0.850 | 1.581 | 1 | 0.210 |
| **Global test** | 5.412 | 9 | 0.800 | 13.742 | 10 | 0.190 |

**
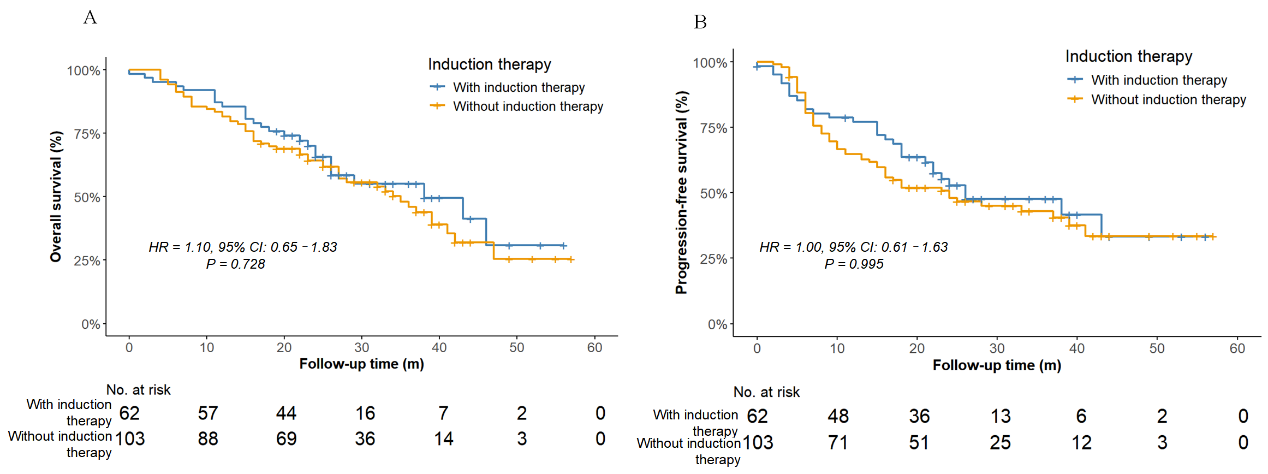
**

**Supplementary Figure 1:** Comparison of survival curves between groups with or without induction therapy after IPTW adjustment. (n = 165) (A) Overall survival. (B) Progression-free survival. HR = hazard ratio, CI = confidence interval.
